# Enhanced Extraction of Polyphenols, Physicochemical Properties, and Microbial Control in *Vitis vinifera* L. Juice Using Ultrasound-Assisted Maceration

**DOI:** 10.3390/molecules30030587

**Published:** 2025-01-27

**Authors:** Magdalena Błaszak, Sabina Lachowicz-Wiśniewska, Ireneusz Kapusta, Małgorzata Szewczuk, Ireneusz Ochmian

**Affiliations:** 1 Department of Bioengineering, West Pomeranian University of Technology in Szczecin, Słowackiego 17, 71-434 Szczecin, Poland; magdalena.blaszak@zut.edu.pl; 2 Department of Medical and Health Sciences, Calisia University, 4 Nowy Świat Street, 62-800 Kalisz, Poland; s.lachowicz-wisniewska@uniwersytetkaliski.edu.pl; 3 Department of Food Technology and Human Nutrition, Rzeszów University, Zelwerowicza 4 Street, 35-601 Rzeszów, Poland; ikapusta@ur.edu.pl; 4 Department of Monogastric Animal Sciences, West Pomeranian University of Technology in Szczecin, 29 Klemensa Janickiego Street, 71-270 Szczecin, Poland; malgorzata.szewczuk@zut.edu.pl; 5 Department of Horticulture, West Pomeranian University of Technology in Szczecin, Słowackiego 17 Street, 71-434 Szczecin, Poland

**Keywords:** polyphenols, color, grape juice, maceration, ultrasounds, decontamination

## Abstract

Polyphenols are essential bioactive compounds that contribute to the nutritional and sensory properties of grape juice and wine. This study investigates the impact of ultrasound-assisted maceration (UAM) compared to traditional maceration (TM) techniques, under both warm and cold conditions, on the polyphenol content, physicochemical properties, and microbial counts of juice from *Vitis vinifera* L. Ultrasound-assisted maceration significantly enhanced the extraction of polyphenols, including anthocyanins, flavonols, flavan-3-ols, and stilbenes, within a shorter processing time. The total polyphenol content increased up to 689.3 mg/L under UAM, while TM required extended maceration periods to achieve comparable results. In addition to polyphenol enrichment, UAM resulted in improved physicochemical properties, including higher extract content (% Brix) and increased turbidity (NTU), with minimal impact on pH and acidity levels. Microbial counts in juice remained low under UAM, indicating that this method may also have antimicrobial benefits due to the cavitation effects of ultrasound. Conversely, TM under warm conditions led to a reduction in extract content and nitrogen availability due to fermentation processes initiated during prolonged maceration. The findings highlight that UAM is a highly efficient technique for enhancing the polyphenol profile of grape juice while preserving key physicochemical parameters and microbial decontamination. This study provides valuable insights for the beverage industry, suggesting that UAM can be a sustainable and time-efficient alternative to traditional maceration methods for producing high-quality grape-based beverages.

## 1. Introduction

Consumers are seeking organic products with reduced preservative content [1,2]. Nuclear and UV-C radiation and ozonation have been investigated as alternatives to sulfuring juices, musts, and wines [3,4,5]. However, ozonation yields ambiguous results, as the effectiveness of ozonation varies depending on yeast strains and experimental conditions. Nuclear radiation is very effective and simple to apply but is currently inaccessible to wine producers due to legal constraints and the scope of permitted use [6]. Therefore, we decided to test cavitation phenomena for removing microorganisms from grape juice. However, besides the decontamination effect, we also investigated the physicochemical properties of must subjected to the action of sound waves.

The working hypothesis assumes a positive influence of sound waves on intensifying the extraction of health-promoting components, deepening the color of the must. The mechanism of action of ultrasound is based on rupturing cells from within due to the formation of gas bubbles (cavitation) within them [7]. In places where the pressure is lowest, air bubbles form, which disappear as the pressure increases, disturbing cellular activity. The energy of ultrasonic waves is absorbed by the liquid, thus generating “acoustic wind”. Large pressure differences and turbulence in the liquid occur. These phenomena cause physical damage, weakening, or rupture of cell structure layers in various organisms [7,8]. The collapse of bubbles can cause the formation of small areas with very high temperatures (5500 °C) and pressure (50 MPa), which can locally damage the cellular structures of microorganisms [9]. The implosion of bubbles and the formation of local hot spots lead to the homolytic cleavage of water molecules and the formation of free radicals (hydroxyl and hydrogen atom). Radicals oxidize compounds present in the liquid and react with each other and with other gases [10,11].

Generally, it can be indicated that Gram-negative bacteria (e.g., *Escherichia*, *Salmonella*, *Pseudomonas*) are more sensitive to ultrasound than Gram-positive bacteria [12]. However, the effect of the action is very diverse and depends on the species of microorganism, the matrix subjected to treatments, and the parameters of the process (power, frequency, exposure time) [13]. To increase the effectiveness of microorganism inactivation, combinations of factors in “multiple hurdle technology” are often used, such as temperature, ultrasound, chemical modification of the matrix, and lytic enzymes [12]. There is a danger that cavitation will also have a destructive effect on valuable bouquet components, which would limit the practical use of ultrasound for must maceration.

The use of ultrasound may be useful/indicated for grape varieties with lower polyphenol potential, such as Pinot Noir. Polyphenols, alongside sugars and organic acids, mainly determine the taste and color of wine. They are also the main antioxidants present in grapes and wine. Their content in fruits is influenced by many factors: the cultivar but also the growing conditions. To improve color and maximize the extraction of phenolic compounds, especially anthocyanins from the skins, a longer maceration period is recommended [14,15].

The primary aim of this study was to evaluate the effectiveness of ultrasound-assisted maceration (UAM) in enhancing the extraction of polyphenols, controlling microbial counts, and improving the physicochemical quality parameters of grape must. This research focused on determining the optimal ultrasound exposure time to maximize polyphenol enrichment while reducing naturally occurring microflora, offering an alternative to sulfuring. Additionally, this study compared UAM with traditional maceration techniques (warm and cold) to assess their relative impact on polyphenol content, microbial stability, and must quality parameters, such as pH, acidity, and extract content. This comprehensive approach aimed to highlight UAM as a sustainable and efficient method for grape must maceration, preserving both the chemical integrity and microbiological safety of the product.

## 2. Results

### 2.1. The Effect of Ultrasound on the Bacteria, Yeast, and Mold

**Figure 1:**
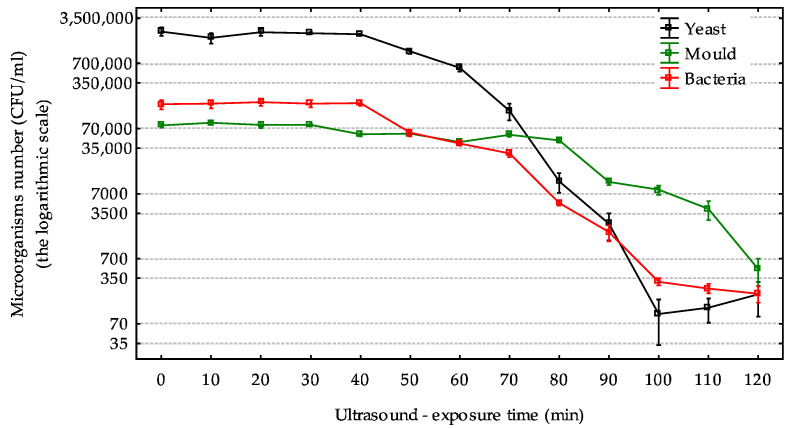
Total microbial number in juice after exposure to ultrasound. The results represent the mean ± SD, *p* < 0.05.

Bacteria were the most susceptible to ultrasound as a significant and lasting decrease in their number in the must was observed earliest. Up to the 40 min of ultrasonic treatment, the count of this group did not change, with bacteria consistently at levels of 140–200 thousand CFU/mL. A drop in count by over 50% was only noted at the 50 min. Further extending the ultrasonic exposure time caused a linear depletion of cells. A 90% reduction occurred by the 90 min of exposure to ultrasound. The bacterial count continued to decrease until the 120 min of the experiment, reaching a level of about 200 CFU/mL (Figure 1).

Yeasts responded to ultrasound similarly to bacteria. At the beginning of the experiment, there were more yeasts than bacteria, approximately 2 million CFU/mL. Until the 40th minute of the experiment, this amount remained at a similar level, and the sound waves did not affect the count of this group. The 50th minute of exposure proved crucial when a statistically significant decrease of about 50% in yeast count was noted. From that point on, the number of yeasts decreased. By the 80th minute, there was a 90% reduction compared to the count at the first measurement. Eventually, the yeast count dropped to the range of 100–200 CFU/mL by the 100th minute of the experiment. Further extending the exposure time of yeasts to ultrasound did not result in any further reduction in their number (Figure 1).

Molds reacted to ultrasound differently than bacteria and yeasts. Molds responded quicker than the other microbial groups to the tested factor, as a significant decrease of about 20% was already noted by the 40th minute. However, in subsequent time intervals up to the 80th minute of the experiment, the count of this group remained at a similar level of about 50 thousand CFU/mL. A 50% reduction in count was noted between the 80th and 90th minute of the experiment. Only by the 120th minute of the experiment was the cell count level comparable to that of bacteria and fungi. A 90% reduction compared to the initial measurement was observed only at the 120th minute (Figure 1).

**Figure 2:**
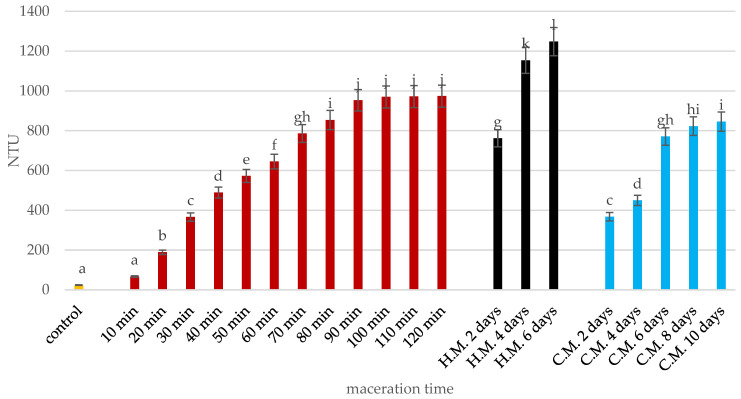
Effect of maceration time and technique on juice turbidity (NTUs). Assessment of the impact of changes in the number of microorganisms that cause turbidity during growth. The yellow bar (K-0) represents the initial juice turbidity (control) before maceration, measured in NTUs (nephelometric turbidity units). The red bars indicate the change in turbidity during ultrasonic maceration over time (10–120 min). The black bars show the turbidity levels during hot maceration (H.M.) at 2, 4, and 6 days. The blue bars represent the turbidity levels during cold maceration (C.M.) at 2, 4, 6, 8, and 10 days. Means, ± SD, followed by the same letter in a row do not differ significantly at *p* = 0.05, according to Tukey’s multiple range test.

From the 80th minute of the experiment, the NTU value is stable (Figure 2). This indicates a reduction in living cells. The cells do not dissappear; they remain in the juice, but they are dead (Figure 1). Therefore, the NTU value does not decrease. In Figure 1, we have confirmation of this fact. All microorganisms die at different rates and to different extents. Turbidity (in NTUs) increased in both methods, albeit with different dynamics (Figure 2). During ultrasonic maceration, turbidity rose rapidly, reaching 974 NTUs after 120 min. During traditional maceration, turbidity increased more gradually, reaching 1248 NTUs under warm conditions after six days and 846 NTUs under cold conditions after ten days. The higher turbidity observed in warm conditions was attributed to the more intensive breakdown of plant tissues, leading to the release of a greater quantity of solid particles. Traditional maceration is carried out for a technologically specified time (usually up to 10 days). It cannot be carried out for too long, as this would reduce the quality of the juice/wine. Moreover, there is no factor limiting the development of microorganisms. Figure 2 shows that the NTU value stabilizes on day 6 of warm maceration. Microorganisms are already at a constant level. In the case of hot maceration, the NTU value increases. It takes a long time for the process to be interrupted due to technological needs. In this case, the number of microorganisms continues to increase (Figure 2).

### 2.2. Influence of Ultrasound on the Physical Characteristics of Musts

**Figure 3:**
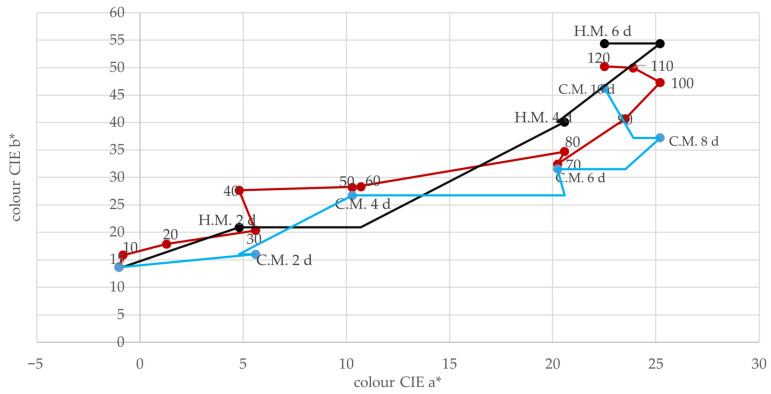
Change in the chromatic color of juice during maceration. The red line (H.M.) represents the change in juice colour during ultrasonic maceration over a period of 0–120 min. The blue line (C.M.) represents the change in juice colour during cold maceration, marked as C.M. The black line (H.M.) represents the change in juice colour during hot maceration, marked as H.M.

Figure 3 illustrates the changes in the color of grape must during ultrasonic maceration (0–120 min) in the CIE a* and b* system. The a* parameter reflects shifts towards redness (positive values) or greenness (negative values), while the b* parameter represents changes towards yellowness (positive values) or blueness (negative values).

At the beginning of ultrasonic maceration, the a* value is low (0.02), indicating no significant color changes. As the process progresses, the a* value increases, demonstrating a faster shift towards redness compared to traditional methods. Simultaneously, the b* parameter shows a sharp increase, particularly after 60 min, indicating a rapid shift towards yellowness. A color similar to that achieved through traditional maceration (6 days of warm maceration or 10 days of cold maceration, represented by a triangle and rectangle, respectively) is reached after approximately 100 min of ultrasonic maceration. These findings confirm the efficiency of ultrasonic maceration in accelerating color development in grape must.

**Figure 4:**
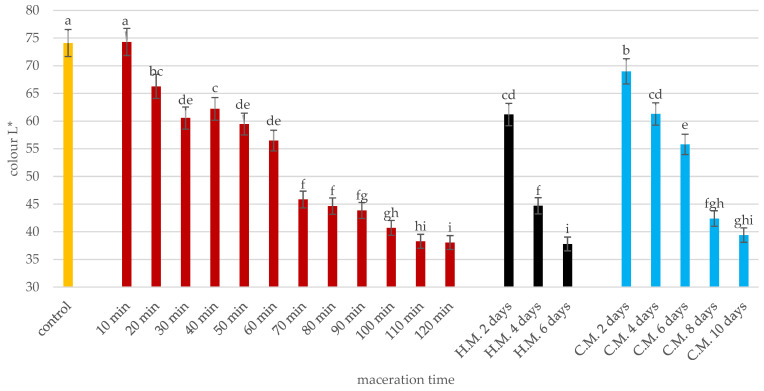
Change in juice lightness (CIE L) during maceration. The yellow bar (control) represents the initial lightness (CIE L*) of the juice before maceration. The red bars show the change in lightness during ultrasonic maceration over time (10–120 min). The black bars indicate the lightness during hot maceration (H.M.) at 2, 4, and 6 days. The blue bars represent the lightness during cold maceration (C.M.) at 2, 4, 6, 8, and 10 days. Means, ± SD, followed by the same letter in a row do not differ significantly at *p* = 0.05 according to Tukey’s multiple range test.

Statistical analysis reveals that in the initial phase (up to approximately 20 min), the differences in L* values are not significant, reflecting minimal variation in color between the samples. However, after 20 min, ultrasonic maceration causes a rapid and statistically significant decrease in the L* parameter. Notably, the L* value achieved after approximately 110 min of ultrasonic maceration becomes comparable to the values obtained after several days of traditional maceration (warm for 6 days or cold for 10 days). These results demonstrate the efficiency of ultrasonic maceration in accelerating color development, significantly shortening the time needed to achieve similar results to traditional methods. Statistical differences marked on the figure emphasize these observations (Figure 4).

### 2.3. Influence of Ultrasound on the Chemical Constituents of Musts

The color change in the must results from the extraction of polyphenolic compounds, especially anthocyanins, from the grape fruit skins. In the control sample, without ultrasound, the polyphenolic content was 3.15 µg/mL—22 times lower than in must subjected to ultrasonic maceration for 120 min. The most significant retention (82%) occurred after just 20 min of ultrasonic waves, followed by increases of 42%, 47%, and 62% after 30, 40, and 50 min, respectively. After 60 to 90 min, the extraction stabilized at 20%, with a steady 5% increase after 100 min. A total of 37 polyphenolic compounds were identified in the must. Due to the large volume of results, only the major groups of polyphenols are presented in this study. The ‘PN’ cultivar must contained five polyphenolic classes: anthocyanins (76.6%), phenolic acids (6.8%), stilbenes (3.1%), and flavonoids such as flavan-3-ols (13.2%) and flavonols (0.2%).

The content of polyphenolic compounds increased with longer maceration times during ultrasonic-assisted maceration. After 120 min, anthocyanins dominated at 56.2%, followed by flavan-3-ols (33%), phenolic acids (5.4%), stilbenes (4.8%), and flavonols (0.6%). Ultrasonic maceration every 10 min over 120 min significantly increased anthocyanins and phenolic acids, with their content 29.8 and 27.7 times higher than the control.

The most intense extraction of phenolic acids occurred between 20 and 50 min, with increases of 76%, 75%, 65%, and 45%. After this, the extraction rate slowed, with only a 5% increase after 80 min. Anthocyanin retention followed a similar pattern, peaking at 117% after 20 min, with slower growth afterward, stabilizing as seen with phenolic acids.

After 120 min, stilbene retention was 14.1 times higher than the control, with significant increases of 52% and 134% at 30 and 40 min, respectively. Flavan-3-ols and flavonols showed 8.8 and 8.5 times higher retention after 120 min. Flavonol extraction was most intense at 10 and 20 min, with increases of 150% and 100%. Flavan-3-ols increased by 42% at 10, 40, and 70 min, and by 30% at 20 and 50 min, with little change afterward. Importantly, no degradation of polyphenolic compounds was observed during ultrasonic maceration (Table 1).

**Figure 5:**
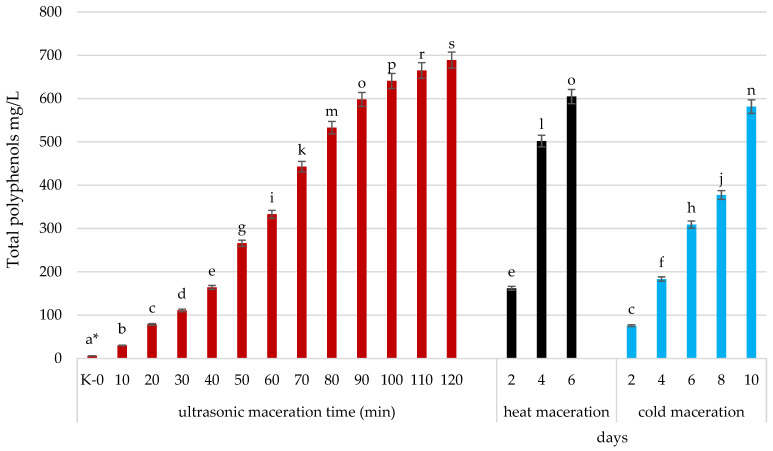
Total polyphenols (mg/L) during ultrasonic and traditional maceration (heat and cold maceration) at selected time intervals. * Means followed by the same letter in rows do not differ significantly at *p* = 0.05 according to Tukey multiple range.

Table 2 and Figure 5 compare the polyphenolic content in musts subjected to traditional cold and warm maceration techniques.

The data show the significant impact of temperature and duration on polyphenol extraction. In warm maceration, polyphenolic content increases progressively over six days, starting at 3.15 mg/L and reaching 604.82 mg/L by day 6. Anthocyanin content surges from 1.77 mg/L to 468 mg/L, demonstrating the effectiveness of heat in extracting pigments. Phenolic acids also rise from 0.17 mg/L to 43.5 mg/L (Figure 4).

The cold maceration results are lower but increase steadily over ten days. Total polyphenol content rises to 581.28 mg/L, and anthocyanin content reaches 495 mg/L by day 10, indicating that prolonged cold maceration effectively extracts these compounds, though slower than warm maceration. Phenolic acids rise to 28.8 mg/L over ten days.

Warm maceration accelerates anthocyanin extraction, peaking at day 6, while cold maceration takes longer but better preserves flavan-3-ols and stilbenes, which are sensitive to heat. This suggests that warm maceration is ideal for quick extraction and intense coloration, while cold maceration may be better for achieving a complex polyphenol profile, particularly for compounds prone to heat degradation. Choosing the right technique depends on the desired chemical and sensory characteristics of the final product.

Significant differences were observed in the dynamics and final values of physicochemical parameters of juice subjected to ultrasonic maceration and traditional maceration under warm and cold conditions.

The soluble solids analysis value (SSA, % Brix) increased steadily during ultrasonic maceration, reaching 22.53% after 120 min, indicating an intensive release of soluble substances from plant tissues. In contrast, traditional maceration exhibited a systematic decline in SSA, particularly under warm conditions, where it dropped from 21.20% to 6.83% over six days. Under cold conditions, the decrease was slower but still substantial, reaching 8.34% after ten days. The reduction in SSA during traditional maceration was attributed to sugar consumption by yeasts during fermentation, which intensified over time.

pH stability was a consistent feature of both methods. In ultrasonic maceration, pH values ranged from 3.14 to 3.25, while in traditional maceration, they varied from 3.17 to 3.26, showing no significant impact on the chemical acidity of the juice.

In summary, ultrasonic maceration was characterized by a rapid increase in extract value and turbidity, along with stable yeast-assimilable nitrogen levels. In contrast, traditional maceration was associated with a decline in extract value and YAN due to fermentation and a more gradual increase in turbidity. These differences highlight the distinct nature of the processes and their potential applications.

The results of the analysis of YAN (yeast-assimilable nitrogen), composed of FAN (Free Amino Nitrogen) and ammonium ions (NH4^+^), reveal significant differences depending on the maceration method and duration. During ultrasound maceration (Table 1), YAN levels increased significantly during the first 20 min, reaching 167 mg/L, and then gradually stabilized within the range of 169–176 mg/L up to 120 min. The increase in YAN was driven by the rise in FAN levels (from 90.11 mg/L to 117.92 mg/L at 110 min) and ammonium ions (NH4^+^), which increased from 44.89 mg/L to 58.08 mg/L. The maximum values of these parameters were observed between 90 and 110 min, indicating an equilibrium state in the release of yeast-assimilable nitrogen compounds. In the case of traditional maceration (Table 2), a decrease in YAN was observed over time, regardless of the temperature of the process. In warm maceration, YAN decreased from an initial value of 135 mg/L to 36 mg/L after six days, whereas in cold maceration, the reduction was slower, with a final value of 58 mg/L recorded after 10 days. This decline in YAN was primarily due to a reduction in FAN levels, which dropped from 90.11 mg/L to 27.03 mg/L in warm maceration and to 35.65 mg/L in cold maceration. Ammonium ion concentrations also decreased, particularly in warm maceration, where they reached 8.97 mg/L after six days, while in cold maceration, they remained at higher levels, ending at 22.35 mg/L after 10 days. These results indicate that ultrasound maceration promotes an increase in the availability of yeast-assimilable nitrogen in the must through the efficient release of FAN and NH4^+^. In contrast, traditional maceration results in a significant depletion of these compounds, particularly under higher temperatures, where yeast metabolic activity is more intense. This suggests that ultrasound maceration may provide a beneficial alternative for enhancing nitrogen availability in musts, supporting more robust fermentation processes.

## 3. Discussion

The fewer spontaneously developing microorganisms in the must, the easier it is to control the winemaking process. The standard procedure involves sulfuring the must to eliminate the native grape microflora, followed by the introduction of noble yeasts Saccharomyces cerevisiae with a predictable biochemical activity profile [16]. However, this method leaves preservative residues at high levels in the final product, and consumers are increasingly reaching for organic and natural wines [1,17]. This study presents an alternative solution to sulfuring the must, namely the use of ultrasound. Ultrasound not only reduced the number of microorganisms in the grapes but also extracted valuable components, polyphenols, from the complex cellular structures of the grapes. The longer the exposure time to the sound waves, the fewer microorganisms remained in the must (Figure 1), and the retention of polyphenolic compounds increased (Table 1). Microorganisms show varied sensitivity to ultrasound. Their reaction depends on several factors including the frequency of the waves, the temperature of the process, exposure time, and the resistance of the microorganisms themselves.

Luo et al. [18] tested the impact of sound waves on various wine-contaminating microorganisms. Ultrasound at a frequency of 24 kHz and 0.2 W/cm^3^ most strongly affected the yeasts *Pichia membranefaciens* and *Saccharomyces cerevisiae*, with only about 40% surviving after 20 min of exposure. On the other hand, *Schizosaccharomyces pombe*, *Hanseniaspora uvarum*, and *Dekkera bruxellensis* showed greater resistance, as their numbers only decreased by about 25% after 20 min of exposure [18]. The use of waves at 24 kHz frequency (400 W, amplitude 100 μm, temperatures of 30 and 40 °C) reduced the population of *Brettanomyces* by an average of 90% and lactic acid bacteria by about 80%; however, unfortunately, this also led to the formation of tar-like and smoky off-odors in wine [19]. Sulfurization of must and wine and other preservation methods usually do not kill all cells, leaving a few percent of live, dormant units, which do not resume activity without sugars and under unfavorable conditions—secondary fermentation [3]. The elimination of 90% of microorganisms from must occurred successively at the 80th minute for yeasts, 90th for bacteria, and 120th minute for molds. As it turns out, there is no single optimal exposure time to ultrasound at which all microorganisms die (Figure 1). However, considering the anaerobic nature of the subsequent stages of winemaking, it can be assumed that advanced mold development will not occur even if reduced quantities of spores remain in the must; as indicated by Novodvorska et al. [20], mold spores are at best able to germinate under aerobic conditions. Therefore, primarily considering bacteria and wild yeasts, it can be suggested that the 90th minute of exposure to sound waves may be the optimal time and have potential significance for the wine industry. Of course, due to the laboratory range of our studies, a deeper analysis of microorganism behavior on a quarter, half, and technical scale would be required [21]. Concurrently with the elimination of microorganisms, the color and chemical composition of the must changed (Figure 1, Figure 2, Figure 3 and Figure 4). These studies indicated that the longer the exposure time to ultrasonic waves of the must, the higher the retention of individual classes of polyphenolic compounds, where the intensity of growth strictly depended on the duration of the extraction (Table 1, Figure 4). However, this process did not indicate degradation of polyphenolic compounds but only affected retention. Effective extraction of individual groups of polyphenolic compounds is an important process, as these compounds exhibit a range of health-promoting properties, including antioxidant effects [22], and also shape the quality characteristics of finished wines [23]. Some of the more important compounds are anthocyanins, in part due to their intense color in must, and their content depends on, among other things, the method of extraction, processing conditions, grape fruit cultivar, etc. [24]. The maceration stage is one of the key stages in the winemaking process, and it aims, among other things, to extract polyphenolic compounds including anthocyanins mainly from the grape fruit skin [24,25,26]. Ultrasound affects the loosening of the cellular structure of the plant and tearing of the tissue, especially the fruit skin, causing more efficient extraction of these compounds, which is also confirmed by studies by Lieu et al. [27], who indicated that the use of ultrasound compared to enzymatic treatment increased the overall content of polyphenols in grape juice after maceration affecting the structure of the grape fruits. Ultrasonic maceration leads to the intensification of polyphenol extraction from grape fruits and contributes to intense color changes in juice and wine [24].

Indeed, the highest concentration of various classes of polyphenolic compounds, including primarily anthocyanins, is located in the grape skins [22,28,29]. Moreover, there is no evidence that ultrasonic treatment during pre-fermentation maceration modifies the sensory or olfactometric profile of wines [30]. The use of ultrasound has also enabled the production of rosé wines with high color intensity and intense aroma in a very short time, limiting wine oxidation [31]. Dos Santos Lima et al. [32] tested different maceration conditions on an industrial scale and found that they did not significantly affect the content of anthocyanins. Conversely, Tiwari et al. [33] demonstrated that ultrasound application led to significant retention of anthocyanins, consistent with observations in this study. However, Hasan et al. [34] reported a decreasing concentration of anthocyanins after the application of ultrasound waves compared to control samples. Tao et al. [35] conducted an extraction with wine lees assisted by ultrasound waves at a frequency of 40 kHz and found that under optimal conditions, ultrasound extracted significantly higher concentrations of total polyphenols and total anthocyanins. This highlights the effectiveness of enhancing the extraction process with ultrasound in terms of retaining bioactive compounds.

The application of such treatment to grape fruits during the wine aging process showed positive sensory-level changes [30]. Meanwhile, Bautista-Ortín et al. [36] used high-power ultrasound in the process of red wine vinification and noted that the exposure to ultrasonic waves, compared to untreated samples, effectively led to a significant increase in the overall content of polyphenols (2.3 times more), anthocyanins (27 times more), and phenolic acids (1.8 times more), improving the final quality of red wines. On the other hand, Celotti et al. [37] analyzed the impact of high-power ultrasound (20 kHz) treatments on the stability of total polyphenols and anthocyanins in red young wines. It was proven that ultrasound with amplitudes of 41 and 81% did not affect the degradation of polyphenolic compounds and anthocyanins, but influenced the degradation of tannins, likely in favor of flavan-3-ols. This could be linked to the phenomenon of depolymerization, which increased the amount of monomeric units including catechins [38], a process possibly facilitated by the applied ultrasonic waves [39]. Differences in the content of individual polyphenols may arise from the grape cultivar used, the level of ultrasound amplitude applied, the temperature, or the length of the process [24].

A comparison of the physicochemical parameters presented in Table 1 and Table 2 reveals significant differences between ultrasonic maceration and traditional maceration. Ultrasonic maceration resulted in a rapid increase in extract content (% Brix) within a short time frame, aligning with findings by Puzovic and Mikulic-Petkovsek [40], where ultrasound enhanced the release of soluble components from grape fruits. In contrast, traditional maceration, which spanned several days, demonstrated a decline in extract content due to sugar utilization by yeasts during fermentation.

Clarity, indicating the degree of turbidity or opacity of grape fruit juices, also showed notable differences. Juice turbidity (NTU) increased significantly in both methods, but the rate of increase was faster with ultrasonic maceration. Ultrasonic treatment effectively releases particles from fruit tissues, leading to a rapid rise in turbidity, as observed by Santhirasegaram et al. [41]. In traditional maceration, higher NTU values under warm conditions are consistent with Zhao et al. [42], where elevated temperatures promoted the release of solid particles during maceration. This phenomenon can be attributed to mechanical stress caused by the cavitation-induced collapse of bubbles, which breaks down large macromolecules and particles in the juice [43].

Yeast-assimilable nitrogen (YAN) content also displayed distinct trends. During ultrasonic maceration, YAN levels increased in the early stages, reflecting improved nutrient availability facilitated by this technology. High-power ultrasound can disrupt cell walls and denature juice enzymes, enhancing the accessibility of nutrients [9]. Conversely, traditional maceration showed a decline in YAN levels, particularly under warm conditions, as fermentation progressed. During alcoholic fermentation, yeasts utilize available nitrogen sources such as nitrates, ammonia, and free amino acids for growth and metabolism. As fermentation continues, these compounds are consumed, leading to a reduction in their concentration within the fermenting medium [44].

The processes of maceration and fermentation under controlled conditions had minimal impact on must pH, which is advantageous for sensory quality and product stability. During wine fermentation, pH undergoes only slight changes, which are critical for microbiological stability and the sensory attributes of the final product. In the early stages of alcoholic fermentation, yeast metabolism of sugars produces organic acids such as succinic acid, resulting in a slight decrease in pH. However, Rakonczás et al. [45] demonstrated that skin maceration and fermentation significantly increased potassium content by approximately 30–70%, correlating with a rise in pH by 0.4–0.5 units.

In summary, ultrasonic maceration offers rapid extract release, enhanced turbidity dynamics, and improved nutrient availability compared to traditional methods. Meanwhile, traditional maceration is characterized by prolonged fermentation dynamics, with declining YAN and stable pH values, underlining the distinct advantages and applications of both processes in grape fruit juice and wine production.

## 4. Materials and Methods

### 4.1. Material

This research utilized grapes (*Vitis vinifera* L.) of the Pinot Noir cultivar. The grape fruits were harvested from an organic plantation located near Szczecin in northwestern Poland (53°21′09.8″ N 14°26′23.3″ E). In the Szczecin area and the surrounding northern region, minimal temperatures typically range from −12 °C to −15 °C, corresponding to values typical of zone 7B. The average temperature during the growing season (April–October) between 1951 and 2019 was 14.3 °C, with rainfall averaging approximately 350 mm [46].

### 4.2. Ultrasonic Reactor and Process Parameters

**Figure 6:**
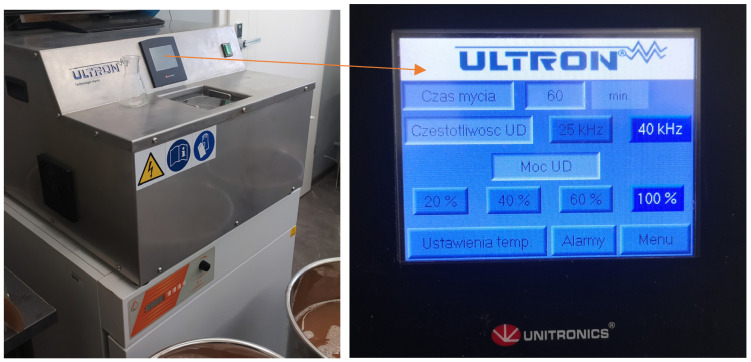
A prototype of a device made especially for the project on the use of physical methods for the preservation of juices, musts, and wines. Possibility to set temperature (cooling), time, power, and frequency of sound waves.

After the grape fruits were harvested, they were destemmed and crushed to break open the skin and expose the pulp. The crushed pulp, prepared each time from 3 kg of destemmed fruits, was then placed in an ultrasound device, namely the ULTRON Unitronics (Dywity, Poland) (Figure 6). The ultrasonic device used was custom-built for our university laboratory by a company specializing in manufacturing pulpers for the industry. This particular model features a cooling option for the fluid and the ability to adjust both power and frequency settings. For the process described, the device was set to a frequency of 40 kHz and a power of 380 W, optimized for the extraction of compounds without damaging the grape fruit tissues excessively.

During ultrasonic irradiation, it is crucial to maintain a controlled temperature, in this case, between 23 and 25 °C, to optimize the extraction processes while preventing undesirable enzyme activities that could spoil the juice. Throughout the ultrasonic treatment, periodic sampling every ten minutes allows monitoring of critical parameters like microbial counts (bacteria, yeasts, and fungi), color, and polyphenol content. This helps in adjusting the process parameters to achieve the desired quality of must.

The ultrasonic waves enhance the extraction of desirable compounds through cavitation. The mechanical action of cavitation increases cell wall permeability, improving the release of phenolics and other compounds into the must. This not only enhances the flavor and color but can also impact the stability of the juice and wine. After the preset time of 120 min, the ultrasonically treated must was processed further according to standard winemaking practices.

Simultaneously, a traditional maceration process of the pulp was conducted. The grape fruits were macerated until a similar color of the must was obtained compared to the one achieved after 120 min of ultrasound-assisted maceration. Traditional maceration was performed at both low temperature (cold maceration at 10–12 °C) and normal temperature (warm maceration at 19–20 °C).

### 4.3. Bacteria, Yeast, Mold

The microbial counts in the juice were assessed using the traditional culturing method. The total number of cultivable bacteria, molds, and yeasts in the juice was tested, without species identification. A dilution series of the material (juice) was prepared and plated onto microbiological media. A classic microbiological procedure for isolating microorganisms from various environmental matrices was followed [47]. Three microbiological media were employed for isolating different microorganisms: Trypticase Soy Agar (BioMaxima, Lublin, Poland) for bacteria, Yeast Extract–Peptone–Glycerol Medium (BTL, Warszawa, Poland) for yeast, and Rose Bengal Agar (BTL Poland) for mold [47,48,49]. Must samples were collected from the working chamber of the device starting from the moment it was turned on. The first sample was taken immediately, and subsequent samples were collected at 10 min intervals (in 4 repetitions). The final samples were collected at the 120th minute of the experiment. A series of dilutions were prepared from the must samples, and the pour plate method was used for microbial enumeration. After an incubation period of 5–7 days at 25 °C, the colony-forming units (CFUs) were counted using the eCount Colony Counter (AlChem, Toruń, Poland).

### 4.4. Polyphenolic Compounds

Polyphenolic compounds were analyzed using the UPLC-PDA-MS/MS Waters ACQUITY system (Waters, Milford, MA, USA), consisting of a binary pump manager, sample manager, column manager, PDA detector, and tandem quadrupole mass spectrometer (TQD) with electrospray ionization (ESI) [50].

### 4.5. Juice Quality

The juice’s turbidity (MTU) was measured using a Lovibond TB211IR (Hach, Iowa, USA) working on the principle of measuring scattered light in the 400–600 nm range.

All physicochemical parameters of the juice/must, including extract content (% Brix), pH, total acidity (g/L), yeast-assimilable nitrogen (YAN, mg/L composed of FAN (Free Amino Nitrogen) and ammonium ions (NH4^+^)), and other chemical properties, were measured using the OenoFoss™ 2 scanner (Foss Analytical A/S, Hillerød, Denmark). This device employs near-infrared (NIR) technology for rapid and precise analysis of samples, allowing for the simultaneous measurement of multiple chemical parameters. All measurements were conducted according to the manufacturer’s instructions, and the results were expressed in the appropriate metric units for each parameter analyzed.

### 4.6. Color

The color parameters were evaluated using the CIE L*a*b* color space, where L* represents lightness (L* = 100 for white and L* = 0 for black), a* represents redness (+a* values) or greenness (−a* values), and b* represents yellowness (+b* values) or blueness (−b* values). Color coordinates were determined according to the 10° standard observer and the D65 standard illuminant. The CIE L*a*b* values were measured using a KonicaMinolta CM-700d spectrophotometer (Chiyoda, Tokio, Japan) [51].

### 4.7. Statistical Analysis

Statistical analyses were performed with Statistica 12.5 (StatSoft Polska, Cracow, Poland). The data were subjected to a *t*-test and multivariate analysis (ANOVA). Mean comparisons were performed using Tukey’s least significant difference (LSD) test; significance was set at *p* < 0.05.

## 5. Conclusions

The use of ultrasound (at a frequency of 40 kHz) undoubtedly accelerated and intensified the maceration process. Compared to traditional maceration techniques (warm and cold), the polyphenol content extracted from the grape fruits after 40 min of ultrasound treatment was comparable to that achieved after two days of warm maceration, and after 90 min, it was comparable to six days. Additionally, ultrasound-assisted maceration (UAM) significantly improved physicochemical properties, such as extract content (% Brix) and turbidity (NTU), while maintaining stable pH and total acidity levels, demonstrating that the ultrasonic process does not compromise the chemical integrity of the juice or must.

From a technological perspective, these results confirm that UAM leads to faster and more intense extraction of polyphenols, improving the color and quality of the must compared to traditional methods. The microbial decontamination effect varied among different groups of microorganisms. Yeasts were the most sensitive to ultrasound, followed by bacteria, with mold fungi being the most resistant. This variability makes it challenging to establish a single effective decontamination time for all microorganisms. However, a time interval of 100–120 min of ultrasound exposure provided the optimal balance, achieving significant decontamination and maximizing the extraction of polyphenols and pigments without compromising the quality of the product.

Moreover, UAM offers a sustainable alternative to chemical preservation, reducing environmental pollution and potential allergen exposure from sulfites. This physical decontamination approach aligns with the growing consumer demand for natural and eco-friendly food processing methods, particularly in mass-produced products such as juices, musts, wines, and dried fruits. Overall, ultrasound-assisted maceration presents a cleaner, more efficient, and sustainable technology for modern food processing industries.

## Figures and Tables

**Table 1:** Polyphenol content and physicochemical properties of juice as a function of ultrasound maceration time.

	Maceration Time (min)												
	K -0	10	20	30	40	50	60	70	80	90	100	110	120
Phenolic acids	0.2 ± 0.0 a *	2.5 ± 0.03 b	4.4 ± 0.05 c	7.8 ± 0.08 d	12.7 ± 0.16 e	18.4 ± 0.22 f	24.8 ± 0.26 g	30.1 ± 0.33 h	37.4 ± 0.44 i	38.2 ± 0.43 i	41.2 ± 0.48 j	43.3 ± 0.49 k	47.1 l ± 0.56
Anthocyanins	1.8 ± 0.02 a	23.8 ± 0.26 b	51.7 ± 0.56 c	78.2 ± 1.02 d	112.9 ± 1.27 e	195.6 ± 2.31 f	249.9 ± 2.91 g	335.1 ± 3.90 h	407.4 ± 4.75 i	466.2 ± 5.22 j	498.0 ± 5.84 k	510.4 ± 5.93 kl	528.0 ± 5.95 l
Flavonols	0.0 ± 0.00 a	0.6 ± 0.01 b	1.0 ± 0.01 c	1.1 ± 0.01 cd	1.2 ± 0.0 cd	1.2 ± 0.01 de	1.3 ± 0.01 de	1.3 ± 0.01 def	1.3 ± 0.01 ef	1.4 ± 0.02 f	1.5 ± 0.02 f	1.7 ± 0.02 g	1.8 ± 0.02 g
Flavan-3-ols	1.0 ± 0.01 a	1.5 ± 0.02 a	19.3 ± 0.24 b	21.1 ± 0.24 b	29.5 ± 0.32 c	39.6 ± 0.43 d	45.0 ± 0.55 d	63.7 ± 0.70 e	69.9 ± 0.83 ef	75.2 ± 0.1 fg	81.3 ± 0.88 g	89.4 ± 1.00 h	91.2 ± 1.01 h
Stilbenes	0.2 ± 0.00 a	1.7 ± 0.03 b	2.3 ± 0.03 c	3.5 ± 0.04 d	8.3 ± 0.11 e	11.3 ± 0.15 f	12.4 ± 0.15 fg	13.3 ± 0.14 g	16.8 ± 0.16 h	17.3 ± 0.17 h	19 ± 0.17 i	20.1 ± 0.26 j	21.2 ± 0.26 k
TOTAL (mg/L)	3.2 a	30.1 b	78.7 c	111.7 d	164.5 e	266.1 f	333.4 g	443.5 h	532.8 i	598.3 j	641 k	664.9 l	689.3 m
SSA (% Brix)	21.20 ± 0.19 a	21.33 ± 0.18 ab	21.46 ± 0.20 abc	21.59 ± 0.20 abc	21.72 ± 0.19 bc	21.85 ± 0.20 cd	21.98 ± 0.18 cde	22.11 ± 0.21 cde	22.24 ± 0.23 cde	22.37 ± 0.22 de	22.50 ± 0.21 e	22.50 ± 0.22 e	22.53 ± 0.23 e
Acidity (g/L)	8.41 ± 0.09 a	8.45 ± 0.10 a	8.42 ± 0.08 a	8.44 ± 0.09 a	8.45 ± 0.10 a	8.54 ± 0.10 a	8.52 ± 0.11 a	8.55 ± 0.09 a	8.54 ± 0.10 a	8.52 ± 0.11 a	8.56 ± 0.10 a	8.55 ± 0.11 a	8.56 ± 0.11 a
NTU	24 ± 0.31 a	67 ± 0.85 b	189 ± 2.35 c	366 ± 4.65 d	489 ± 6.40 e	573 ± 7.09 f	645 ± 8.32 g	786 ± 9.87 h	854 ± 10.92 i	953 ± 12.15 j	970 ± 12.33 j	972 ± 12.28 j	974 ± 12.39 j
pH	3.21 ± 0.04 a	3.23 ± 0.04 a	3.20 ± 0.04 a	3.22 ± 0.04 a	3.25 ± 0.04 a	3.19 ± 0.04 a	3.19 ± 0.04 a	3.20 ± 0.04 a	3.18 ± 0.04 a	3.15 ± 0.04 a	3.15 ± 0.04 a	3.16 ± 0.04 a	3.14 ± 0.04 a
YAN = FAN + NH4^+^ (mg/L)	135 ± 3.17 a	146 ± 3.22 a	167 ± 3.55 b	169 ± 3.71 b	170 ± 3.90 b	169 ± 3.83 b	172 ± 3.81 b	175 ± 3.90 b	173 ± 3.91 b	175 ± 4.02 b	176 ± 3.85 b	176 ± 3.90 b	175 ± 4.04 b
FAN	90.11	97.82	111.89	113.23	113.9	113.23	115.24	117.25	115.91	117.25	117.92	117.92	117.25
NH4^+^	44.89	48.18	55.11	55.77	56.1	55.77	56.76	57.75	57.09	57.75	58.08	58.08	57.75

* Means followed by the same letter in rows do not differ significantly at *p* = 0.05 according to Tukey multiple range. SSA—soluble solids analysis; NTU—turbidity of juice; YAN—yeast-assimilable nitrogen; FAN—Free Amino Nitrogen.

**Table 2:** Polyphenol content and physicochemical properties of juice as a function of time and temperature during traditional maceration.

	Warm Maceration Days				Cold Maceration Days					
	K -0	2	4	6	K -0	2	4	6	8	10
Phenolic acids	0.2 ± 0.0 a *	17.5 ± 0.18 b	36.7 ± 0.39 c	43.5 ± 0.50 d	0.2 ± 0.0 A*	2.4 ± 0.03 B	4.0 ± 0.05 B	12.9 ± 0.14 C	25.6 ± 0.25 D	28.8 ± 0.31 E
Anthocyanins	1.8 ± 0.02 a	134.0 ± 1.50 b	392 ± 4.50 c	468 ± 5.32 d	1.8 ± 0.02 A	68 ± 0.75 B	166 ± 1.84 C	261 ± 3.01 D	305 ± 3.28 E	495 ± 5.49 F
Flavonols	0.0 ± 0.00 a	0.5 ± 0.01 b	1.1 ± 0.01 c	1.6 ± 0.02 d	0.0 ± 0.00 A	0.3 ± 0.00 B	0.4 ± 0.01 B	0.5 ± 0.01 C	1.1 ± 0.02 D	1.5 ± 0.02 E
Flavan-3-ols	1.0 ± 0.01 a	8.9 ± 0.09 b	62.0 ± 0.69 c	75.2 ± 0.79 d	1.0 ± 0.01 A	4.9 ± 0.06 B	12.3 ± 0.11 C	29.0 ± 0.31 D	36.5 ± 0.37 E	42.8 ± 0.48 F
Stilbenes	0.2 ± 0.00 a	1.2 ± 0.01 b	10.3 ± 0.10 c	16.5 ± 0.19 d	0.2 ± 0.00 A	0.2 ± 0.00 A	0.7 ± 0.01 B	5.7 ± 0.06 C	9.3 ± 0.11 D	13.2 ± 0.16 E
TOTAL (mg/L)	3.2 a	162.1 b	502.1 c	604.8 d	3.2 A	75.7 B	183.4 C	309.1 D	377.5 E	581.3 F
SSA (% Brix)	21.20 ± 0.19 a	17.31 ± 0.22 b	11.46 ± 0.15 c	6.83 ± 0.08 d	21.20 ± 0.19 A	19.56 ± 0.23 B	14.70 ± 0.19 C	11.98 ± 0.15 D	9.22 ± 0.12 E	8.34 ± 0.11 E
Acidity (g/L)	8.41 ± 0.09 a	8.34 ± 0.10 a	8.35 ± 0.09 a	8.27 ± 0.09 a	8.41 ± 0.09 A	8.37 ± 0.10 A	8.35 ± 0.11 A	8.34 ± 0.10 A	8.31 ± 0.10 A	8.29 ± 0.10 A
NTU	24 ± 0.31 a	762 ± 8.32 b	1154 ± 12.54 c	1248 ± 13.99 d	24 ± 0.31 A	368 ± 4.23 B	450 ± 5.07 C	771 ± 8.50 D	823 ± 9.32 DE	846 ± 9.53 E
pH	3.21 ± 0.04 a	3.23 ± 0.04 a	3.17 ± 0.04 a	3.19 ± 0.04 a	3.21 ± 0.04 A	3.19 ± 0.04 A	3.18 ± 0.04 A	3.22 ± 0.04 A	3.24 ± 0.04 A	3.26 ± 0.04 A
YAN = FAN + NH4^+^ (mg/L)	135 ± 3.17 a	104 ± 2.62 b	72 ± 1.72 c	36 ± 0.93 d	135 ± 3.17 a	117 ± 2.99 B	98 ± 2.61 C	81 ± 2.11 D	67 ± 1.79 DE	58 ± 1.56 E
FAN	90.11	65.08	39.55	27.03	90.11	79.67	71.03	58.33	45.11	35.65
NH4^+^	44.89	38.92	32.45	8.97	44.89	37.33	26.97	22.67	21.89	22.35

* For explanation, see Table 1. Lowercase letters indicate warm maceration; uppercase letters indicate cold maceration.

## Data Availability

Data are contained within the article.

## References

[1] Grogan K.A. (2015). The value of added sulphur dioxide in French organic wine. Agric. Econ..

[2] Ochmian I., Malinowski R. (2024). Effect of Multi-Year Protection of Grapevines with Copper Pesticides on the Content of Heavy Metals in Soil, Leaves, and Fruit. Agronomy.

[3] Błaszak M., Nowak A., Lachowicz S., Migdał W., Ochmian I. (2019). E-Beam irradiation and ozonation as an alternative to the sulphuric method of wine preservation. Molecules.

[4] Błaszak M., Jakubowska B., Lachowicz-Wiśniewska S., Migdał W., Gryczka U., Ochmian I. (2023). Effectiveness of E-Beam radiation against *Saccharomyces cerevisiae*, *Brettanomyces bruxellensis*, and wild yeast and their influence on wine quality. Molecules.

[5] Pachnowska K., Kochel-Karakulska J., Augustyniak A., Obradović V., Ochmian I., Lachowicz-Wiśniewska S., Kapusta I., Maślana K., Mijowska E., Cendrowski K. (2025). UV-C and Nanomaterial-Based Approaches for Sulfite-Free Wine Preservation: Effects on Polyphenol Profile and Microbiological Quality. Molecules.

[6] Kazakis N.A., Betsou C. (2023). Detection of baby food sterilized with ionizing radiation using thermoluminescence. Eur. Phys. J. Spec. Top..

[7] Zupanc M., Pandur Ž., Stepišnik Perdih T., Stopar D., Petkovšek M., Dular M. (2019). Effects of cavitation on different microorganisms: The current understanding of the mechanisms taking place behind the phenomenon. Ultrason. Sonochem..

[8] Piyasena P., Mohareb E., McKellar R.C. (2003). Inactivation of microbes using ultrasound: A review. Int. J. Food Microbiol..

[9] Chemat F., Khan M.K. (2011). Applications of ultrasound in food technology: Processing, preservation and extraction. Ultrason. Sonochem..

[10] Reisz P., Berdahl D., Christman C.L. (1985). Free radical generation by ultrasound in aqueous and nonaqueous solutions. Environ. Health Perspect..

[11] Yusof N.S.M., Babgi B., Alghamdi Y., Aksu M., Madhavan J., Ashokkumar M. (2016). Physical and chemical effects of acoustic cavitation in selected ultrasonic cleaning applications. Ultrason. Sonochem..

[12] Lauteri C., Ferri G., Piccinini A., Pennisi L., Vergara A. (2023). Ultrasound Technology as Inactivation Method for Foodborne Pathogens: A Review. Foods.

[13] Soro A.B., Oliveira M., O’Donnell C.P., Tiwari B.K. (2021). Ultrasound assisted modulation of yeast growth and inactivation kinetics. Ultrason. Sonochem..

[14] Costa E., da Silva J.F., Cosme F., Jordão A.M. (2015). Adaptability of some French red grape varieties cultivated at two different Portuguese terroirs: Comparative analysis with two Portuguese red grape varieties using physicochemical and phenolic parameters. Int. Food Res. J..

[15] Du Toit W.J., Oberholser A., Preedy V.R. (2014). Processing and impact on antioxidants in beverages. Processing and Impact on Antioxidants in Beverages.

[16] Pateraki C., Paramithiotis S., Doulgeraki A.I., Kallithraka S., Kotseridis Y., Drosinos E.H. (2014). Effect of sulfur dioxide addition in wild yeast population dynamics and polyphenolic composition during spontaneous red wine fermentation from *Vitis vinifera* cultivar Agiorgitiko. Eur. Food Res. Technol..

[17] Francesca N., Sannino C., Settanni L., Corona O., Barone E., Moschetti G. (2014). Microbiological and chemical monitoring of Marsala base wine obtained by spontaneous fermentation during large-scale production. Ann. Microbiol..

[18] Luo H., Schmid F., Grbin P.R., Jiranek V. (2012). Viability of common wine spoilage organisms after exposure to high power ultrasonics. Ultrason. Sonochem..

[19] Gracin L., Jambrak A.R., Juretić H., Dobrović S., Barukčić I., Grozdanović M., Smoljanić G. (2016). Influence of high power ultrasound on *Brettanomyces* and lactic acid bacteria in wine in continuous flow treatment. Appl. Acoust..

[20] Novodvorska M., Stratford M., Blythe M.J., Wilson R., Beniston R.G., Archer D.B. (2016). Metabolic activity in dormant conidia of *Aspergillus niger* and developmental changes during conidial outgrowth. Fungal Genet. Biol..

[21] Efara E., Marquis F., Tremblay A. (2019). Scaling Up Biotechnological Chemical Processes: A Better Alternative to the Traditional Develop-Then-Scale Model. Ind. Biotechnol..

[22] Oszmiański J., Lachowicz S. (2016). Effect of the production of dried fruits and juice from chokeberry (*Aronia melanocarpa* L.) on the content and antioxidative activity of bioactive compounds. Molecules.

[23] Setford P.C., Jeffery D.W., Grbin P.R., Muhlack R.A. (2017). Factors affecting extraction and evolution of phenolic compounds during red wine maceration and the role of process modeling. Trends Food Sci. Technol..

[24] Guler A. (2023). Effects of different maceration techniques on the colour, polyphenols, and antioxidant capacity of grape juice. Food Chem..

[25] Kapusta I., Cebulak T., Oszmiański J. (2018). Characterization of Polish wines produced from the interspecific hybrid grapes grown in south-east Poland. Eur. Food Res. Technol..

[26] Ochmian I., Przemieniecki S.W., Błaszak M., Twarużek M., Lachowicz-Wiśniewska S. (2024). Antioxidant, Nutritional Properties, Microbiological, and Health Safety of Juice from Organic and Conventional Solaris Wine (*Vitis vinifera* L.) Farming. Antioxidants.

[27] Lieu L.N., Le V.V.M. (2010). Application of ultrasound in grape mash treatment in juice processing. Ultrason. Sonochem..

[28] Lachowicz S., Wojdyło A., Chmielewska J., Oszmiański J. (2017). The influence of yeast type and storage temperature on content of phenolic compounds, antioxidant activity, colour and sensory attributes of chokeberry wine. Eur. Food Res. Technol..

[29] Aleixandre-Tudo J.L., Buica A., Nieuwoudt H., Aleixandre J.L., du Toit W. (2017). Spectrophotometric analysis of phenolic compounds in grapes and wines. J. Agric. Food Chem..

[30] Sánchez-Córdoba C., Durán-Guerrero E., Castro R. (2021). Olfactometric and sensory evaluation of red wines subjected to ultrasound or microwaves during their maceration or ageing stages. LWT.

[31] Labrador Fernández L., Pérez-Porras P., Díaz-Maroto M.C., Gómez-Plaza E., Pérez-Coello M.S., Bautista-Ortín A.B. (2023). The technology of high-power ultrasound and its effect on the color and aroma of rosé wines. J. Sci. Food Agric..

[32] Dos Santos Lima M., Dutra M.D.C., Toaldo I.M., Corrêa L.C., Pereira G.E., de Oliveira D., Ninow J.L. (2015). Phenolic compounds, organic acids and antioxidant activity of grape juices produced in industrial scale by different processes of maceration. Food Chem..

[33] Tiwari B.K., Patras A., Brunton N., Cullen P.J., O’Donnell C.P. (2010). Effect of ultrasound processing on anthocyanins and color of red grape juice. Ultrason. Sonochem..

[34] Hasan M.M., Yun H.K., Kwak E.J., Baek K.H. (2014). Preparation of resveratrol-enriched grape juice from ultrasonication treated grape fruits. Ultrason. Sonochem..

[35] Tao Y., Wu D., Zhang Q.A., Sun D.W. (2014). Ultrasound-assisted extraction of phenolics from wine lees: Modeling, optimization and stability of extracts during storage. Ultrason. Sonochem..

[36] Bautista-Ortín A.B., Jiménez-Martínez M.D., Jurado R., Iniesta J.A., Terrades S., Andrés A., Gómez-Plaza E. (2017). Application of high-power ultrasounds during red wine vinification. Int. J. Food Sci. Technol..

[37] Celotti E., Stante S., Ferraretto P., Román T., Nicolini G., Natolino A. (2020). High power ultrasound treatments of red young wines: Effect on anthocyanins and phenolic stability indices. Foods.

[38] Morera J., Bartoli E., Combalia F., Castell J., Sorolla S. (2010). Study of the application of ultrasound in vegetable tannage. J. Am. Leather Chem. Assoc..

[39] Zhang Q.A., Shen Y., Fan X.H., Garcia Martin J.F. (2016). Preliminary study of the effect of ultrasound on physicochemical properties of red wine. CyTA J. Food.

[40] Puzovic A., Mikulic-Petkovsek M. (2024). Comparative Evaluation of Conventional and Emerging Maceration Techniques for Enhancing Bioactive Compounds in Aronia Juice. Foods.

[41] Santhirasegaram V., Razali Z., Somasundram C. (2013). Effects of thermal treatment and sonication on quality attributes of Chokanan mango (*Mangifera indica L.*) juice. Ultrason. Sonochem..

[42] Zhao W., Hua J., Wang R., Li S., Shi J., Zhang Z. (2022). Effects of different pectinase maceration pre-treatments on physicochemical properties of raspberry juice and wine. Czech J. Food Sci..

[43] Demirdöven A., Baysal T. (2008). The use of ultrasound and combined technologies in food preservation. Food Rev. Int..

[44] Beltran G., Rozes N., Mas A., Guillamon J.M. (2007). Effect of low-temperature fermentation on yeast nitrogen metabolism. World J. Microbiol. Biotechnol..

[45] Rakonczás N., Andrási D., Murányi Z. (2015). Maceration affects mineral composition and pH of wines. Int. J. Hortic. Sci..

[46] Figiel-Kroczyńska M., Ochmian I., Lachowicz S., Krupa-Małkiewicz M., Wróbel J., Gamrat R. (2021). *Actinidia* (mini kiwi) fruit quality in relation to summer cutting. Agronomy.

[47] Speranza B., Campaniello D., Petruzzi L., Sinigaglia M., Corbo M.R., Bevilacqua A. (2019). Preliminary Characterization of Yeasts from Bombino Bianco, a Grape Variety of Apulian Region, and Selection of an Isolate as a Potential Starter. Fermentation.

[48] Ottow J.C.G. (1972). Rose Bengal as a Selective Aid in the Isolation of Fungi and Actinomycetes from Natural Sources. Mycologia.

[49] McCaig A.E., Grayston S.J., Prosser J.I., Glover L.A. (2001). Impact of cultivation on characterization of species composition of soil bacterial communities. FEMS Microbiol. Ecol..

[50] Lachowicz-Wiśniewska S., Kapusta I., Stinco C.M., Meléndez-Martínez A.J., Bieniek A., Ochmian I., Gil Z. (2021). Distribution of polyphenolic and isoprenoid compounds and biological activity differences between in the fruit skin + pulp, seeds, and leaves of new biotypes of *Elaeagnus multiflora* Thunb. Antioxidants.

[51] Chełpiński P., Ochmian I., Forczmański P. (2019). Sweet cherry skin colour measurement as a non-destructive indicator of fruit maturity. Acta Univ. Cibiniensis. Ser. E Food Technol..

